# Human Metabolome-derived Cofactors Are Required for the Antibacterial Activity of Siderocalin in Urine*[Fn FN2]

**DOI:** 10.1074/jbc.M116.759183

**Published:** 2016-10-25

**Authors:** Robin R. Shields-Cutler, Jan R. Crowley, Connelly D. Miller, Ann E. Stapleton, Weidong Cui, Jeffrey P. Henderson

**Affiliations:** From the ‡ Division of Infectious Diseases, Department of Medicine,; the § Center for Women's Infectious Diseases Research, and; the ¶ Department of Internal Medicine, Washington University School of Medicine, St. Louis, Missouri 63110,; the ‖ Department of Medicine, Division of Allergy and Infectious Diseases, University of Washington, Seattle, Washington 98195, and; the ** Department of Chemistry, Washington University, St. Louis, Missouri 63130

**Keywords:** Escherichia coli (E. coli), host-pathogen interaction, infectious disease, iron, mass spectrometry (MS), siderophore, NGAL, lipocalin 2, siderocalin, urinary tract infection

## Abstract

In human urinary tract infections, host cells release the antimicrobial protein siderocalin (SCN; also known as lipocalin-2, neutrophil gelatinase-associated lipocalin, or 24p3) into the urinary tract. By binding to ferric catechol complexes, SCN can sequester iron, a growth-limiting nutrient for most bacterial pathogens. Recent evidence links the antibacterial activity of SCN in human urine to iron sequestration and metabolomic variation between individuals. To determine whether these metabolomic associations correspond to functional Fe(III)-binding SCN ligands, we devised a biophysical protein binding screen to identify SCN ligands through direct analysis of human urine. This screen revealed a series of physiologic unconjugated urinary catechols that were able to function as SCN ligands of which pyrogallol in particular was positively associated with high urinary SCN activity. In a purified, defined culture system, these physiologic SCN ligands were sufficient to activate SCN antibacterial activity against *Escherichia coli*. In the presence of multiple SCN ligands, native mass spectrometry demonstrated that SCN may preferentially combine different ligands to coordinate iron, suggesting that availability of specific ligand combinations affects *in vivo* SCN antibacterial activity. These results support a mechanistic link between the human urinary metabolome and innate immune function.

## Introduction

Iron is an essential micronutrient for nearly all microorganisms, and its acquisition is a critical virulence-associated activity for numerous medically important pathogens (1, 2). Humans possess elaborate systems that limit the accessibility of iron to pathogens (1, 3, 4). Among these innate immune defenses is siderocalin (SCN^3^; also known as lipocalin-2 or neutrophil gelatinase-associated lipocalin), a ∼25-kDa soluble protein of the lipocalin family notable for a characteristic eight-stranded antiparallel β-barrel fold that defines a binding site, or calyx (5). Compared with other lipocalin proteins, SCN is distinguished by a shallow, positively charged calyx with three distinct binding pockets (5–7). The SCN calyx does not chelate Fe(III) alone but instead as a complex with catechol ligands, each of which binds the protein through one of three calyx pockets (5, 8). The resulting SCN-ferric catechol complex shields iron in a form that is poorly accessible to bacteria.

The prototypical SCN ligand is enterobactin, a secreted microbial iron chelator (siderophore) that chelates ferric ions with extraordinary affinity using three interconnected catechols (5, 9–11). In culture medium, SCN can inhibit growth of urinary pathogenic *Escherichia coli* that use enterobactin as their sole siderophore, likely through sequestration of Fe(III)-enterobactin (5, 12, 13). However, when these same *E. coli* strains are grown in human urine, enterobactin becomes necessary to *resist* SCN inhibitory activity, suggesting that urinary constituents change the interaction between SCN and enterobactin (14). Because SCN reaches high levels during human urinary tract infections, these interactions may influence infection pathogenesis (14–16).

Human urinary effects on SCN antibacterial activity exhibit marked individual differences (14). In urine from “restrictive” subjects, SCN inhibits bacterial growth in an enterobactin-sensitive manner. In urine from “permissive” subjects, neither SCN nor bacterial enterobactin production exerts prominent effects on *E. coli* growth. Compared with permissive urine, restrictive urine is associated with a higher pH and distinctive aromatic metabolites. These urinary metabolites are catechols and other aromatic compounds that have been conjugated to sulfate, most likely in the liver. Although catechols can act as Fe(III)-chelating SCN ligands, their sulfated forms generally do not, suggesting that these aryl sulfates do not directly interact with SCN. Nevertheless, greater SCN activity at pH above 6.5 is consistent with pH optima for ferric catechol complex formation and binding within the calyx (8, 17). We therefore hypothesized that the SCN activity-associated metabolites are biomarkers for unconjugated SCN ligands, which form stable ferric complexes in the conditions of restrictive urine (14).

In this study, we show that SCN and enterobactin are present simultaneously during human *E. coli* UTIs and seek to identify physiologically relevant SCN ligands through direct analysis of human urine. A biophysical screen, intrinsic tryptophan fluorescence quenching, and mass spectrometric analyses identified a series of endogenous SCN ligands in human urine of which unconjugated pyrogallol and caffeic acid were associated with high urinary SCN activity. Although defined medium conditions containing a single SCN ligand were sufficient to activate SCN antibacterial activity, native mass spectrometry revealed that SCN can use a specific ligand combination to chelate Fe(III) when multiple ligands are available. Together, these studies identify free catechol metabolites in humans that potentiate innate antibacterial immunity by acting as Fe(III)-chelating siderocalin cofactors.

## Results

### 

#### 

##### Enterobactin and SCN Coexist during Clinical E. coli UTI

**FIGURE 1.**
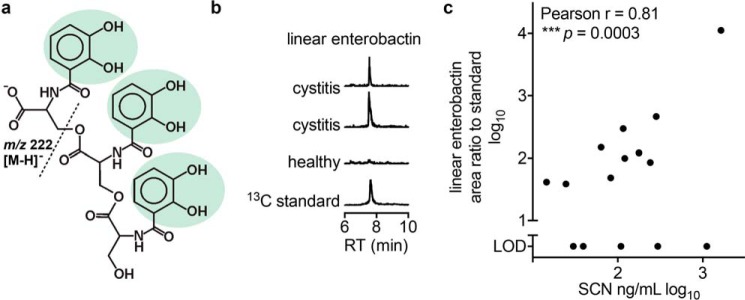
**Urinary enterobactin and SCN in human urinary tract infections caused by *E. coli*.** *a*, structure of the *E. coli* siderophore linear enterobactin. The three catechol groups are highlighted in *green*, and the site of MS/MS cleavage to yield the *m*/*z* 222 product ion is denoted with a *dashed line. b*, LC-MS/MS chromatograms for urinary linear enterobactin are shown from two representative *E. coli* cystitis urines and a healthy control urine. An LC-MS/MS chromatogram for the ^13^C internal standard is shown at the *bottom. c*, log-log plot of relative urinary enterobactin concentration *versus* SCN concentration in urines from 15 *E. coli* cystitis patients (*dots*). A positive association is indicated by Pearson correlation (***, *p* = 0.0003). *LOD*, limit of detection for enterobactin signal. *RT*, retention time.

To determine whether enterobactin and SCN are simultaneously present in urine during human urinary tract infections, we analyzed urine specimens from uncomplicated *E. coli* cystitis patients with stable isotope dilution liquid chromatography (LC)-MS/MS (Fig. 1*a*) and ELISA. Linear enterobactin was detectable in 10 of 15 cystitis urine specimens but was undetectable in healthy donors without urinary symptoms or bacteriuria (Fig. 1*b*). SCN was detected in all cystitis specimens and was positively associated with enterobactin (Fig. 1*c*). To our knowledge, this is the first report to directly demonstrate bacterial enterobactin production during a human infection. Moreover, the presence of siderocalin in the same specimens raises the possibility that enterobactin interactions with this protein are physiologically relevant.

##### Differential Scanning Fluorimetry (DSF) Detects Urinary SCN Ligands

**FIGURE 2.**
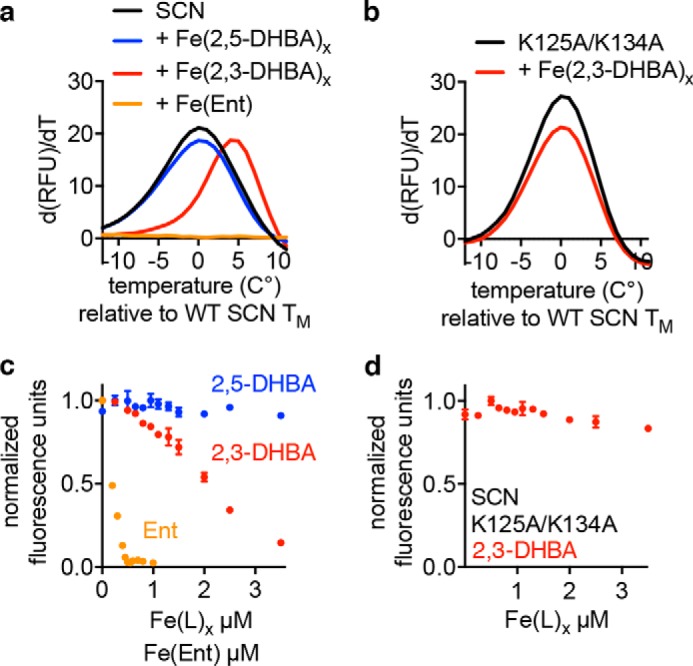
**DSF detects SCN ferric complexes.** The established SCN ligands enterobactin (*Ent*) and 2,3-DHBA (30 μm) alter the *T_m_* of SCN (3 μm), whereas 2,5-DHBA does not (*a*). Plots show the first derivative of relative fluorescence units (*RFU*) as a function of temperature, so a lack of melting transition appears as a flat line. Mutation of two critical lysines in the SCN calyx (K125A/K134A) disrupts the calyx charge and structure; in contrast to WT SCN, the *T_m_* of the mutant protein is not affected by ferric 2,3-DHBA (*b*). FQ mirrors these results with quenching observed in the presence of ferric enterobactin and ferric 2,3-DHBA but not ferric 2,5-DHBA (*c*). No quenching is seen for SCN K125A/K134A (500 nm) by ferric 2,3-DHBA (*d*). *Error bars* represent S.D.

Previous data indicate that urinary SCN uses urinary aryl cofactors to chelate iron in a stable complex (8, 14, 18), suggesting that a binding assay could be used to identify these putative SCN-binding urinary molecules. Although intrinsic tryptophan fluorescence quenching (FQ) has been used extensively to examine SCN binding (5, 8, 19), its susceptibility to spectroscopic interference prevents its use in chemically complex, urine-derived specimens. We therefore developed a different screening assay using a combined LC-DSF approach. DSF identifies chromatographic fractions containing SCN ligands through the thermal stabilization they confer to SCN during the melting transition. Because DSF measures protein denaturation with a high quantum yield fluorescent dye (SYPRO® Orange) and analyzes the first derivative of the melting curve, interference from the chemically complex urinary background is minimized (20). With DSF, the prototypical SCN ligands enterobactin and 2,3-dihydroxybenzoic acid (2,3-DHBA) exhibited altered melting transitions, whereas the non-binding 2,3-DHBA isomer 2,5-dihydroxybenzoic acid (gentisic acid) exhibited no binding signal (Fig. 2*a*) (21). Enterobactin binding appears to prevent SCN from melting in our assay, consistent with previous reports describing high SCN-enterobactin complex stability (5). 2,3-DHBA did not alter the melting transitions of the non-binding SCN calyx mutant where two critical lysine residues have been mutated to alanines (SCN K125A/K134A; Fig. 2*b*) (8). DSF positivity corresponded to the analogous FQ signal with the same reagents (Fig. 2, *c* and *d*). These results support DSF as a feasible screening tool for urinary SCN ligands.

##### SCN Ligands Are Present in Uninfected Human Urine

**FIGURE 3.**
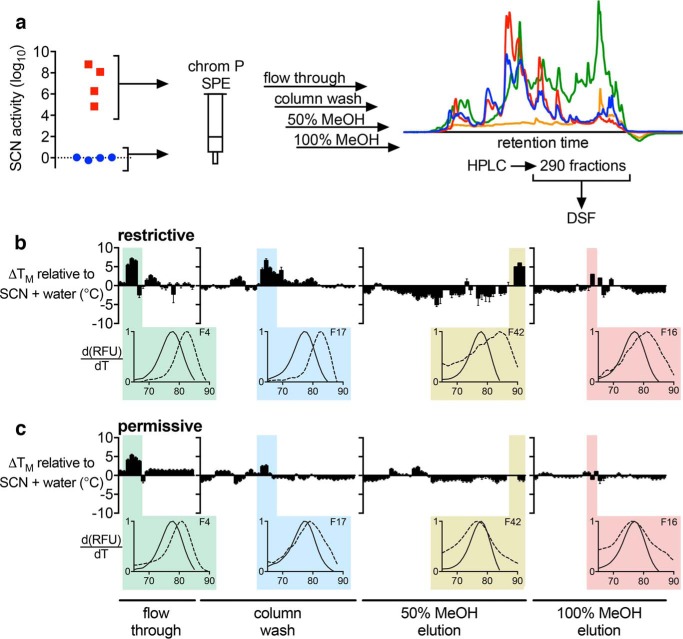
**DSF screening reveals multiple iron-chelating SCN ligands in restrictive human urine.** *a*, urine fractionation scheme. Four urine specimens supporting high SCN activity (restrictive; *red squares*) and four supporting low activity (permissive; *blue circles*) were pooled and extracted by Chrom P SPE prior to HPLC fractionation. An example chromatogram is shown for the 50% methanol elution where *blue*, *red*, *green*, and *orange* traces show absorbance at 240, 280, 350, and 435 nm, respectively. DSF analysis of HPLC fractions (reconstituted to ∼80-fold concentration over starting urine) was conducted with 3.2 μg/ml additional FeCl_3_ to identify ferric siderocalin complexes in the pooled restrictive (*b*) or permissive (*c*) urine fractions. *Columns* show the change in *T_m_* (Δ*T_m_*) for each fraction relative to solvent control. HPLC fractions are presented consecutively as *bars* along the *x axis*; *error bars* represent S.E.M. Fractions of interest from each SPE elution are highlighted, and a representative DSF plot is shown *below* (fraction in *dotted line*; SCN control in *solid line*).

**FIGURE 4.**
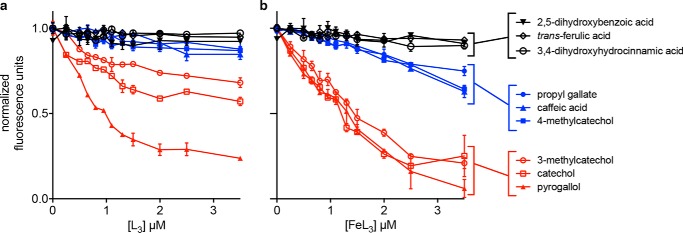
**Direct measurement of SCN binding validates urinary ligands.** SCN binding was directly observed using intrinsic tryptophan fluorescence quenching. GC-MS candidates identified from DSF-positive fractions were added to SCN (500 nm) at a range of concentrations in the absence (*a*) or presence (*b*) of additional FeCl_3_ at a 1:3 molar ratio to the candidate. Candidate metabolites cluster into three binding groups: in *black* are metabolites that do not bind SCN even at large molar excess; in *blue* are intermediate ligands that bind moderately, and most only in the presence of added iron; and in *red* are preferred ligands that show some binding in the absence of supplemental iron and show the highest binding and nearly complete quenching when present in excess as ferric complexes. *Error bars* represent S.D.

We hypothesized that urine supporting greater SCN antimicrobial activity would have higher SCN ligand content. To test this, we applied LC-DSF to human urine specimens (Fig. 3*a*). Two pooled urine specimens were prepared and screened (Table 1): *restrictive* urine from four subjects that supports high SCN antimicrobial activity and *permissive* urine from four subjects that supports minimal SCN activity. Fractions from both urine pools increased SCN melting temperature (*T_m_*) values in the presence of ferric ions (Fig. 3, *b* and *c*, and supplemental Fig. S1). The restrictive urine pool exhibited more *T_m_*-shifting fractions and greater Δ*T_m_* values than the permissive urine pool (Fig. 3, *b versus c*). Metabolomic profiling of active fractions by gas chromatography-mass spectrometry (GC-MS) revealed multiple urinary SCN ligand candidates. Where possible, we confirmed the metabolite identities assigned by spectral matching through comparison with commercially available reference compounds. Metabolites that were enriched in DSF-positive fractions relative to adjacent DSF-negative fractions are listed in Table 2. To determine which metabolites are SCN ligands, we assessed the 13 commercially available candidates (including both 3- and 4-methyl isomers of methylcatechol) using DSF (supplemental Fig. S2) and FQ (Fig. 4) binding assays. Of these metabolites, six changed the SCN *T_m_*, and five of those also exhibited an FQ binding signature (Fig. 4). Only 3,4-dihydroxyhydrocinnamic acid exhibited discrepant results with activity in the DSF but not the FQ assay. The five metabolites with both DSF and FQ binding signals, pyrogallol, caffeic acid, 3-methylcatechol, 4-methylcatechol, and propyl gallate, all contain a catechol moiety, consistent with the chemical group's Fe(III) chelation, SCN binding (8, 18, 22), and urinary SCN activity (14) associations. These results identify a population of iron-chelating SCN ligands in human urine and suggest that qualitative and quantitative differences among these may distinguish restrictive and permissive urine. Of note, none of these metabolites are sulfate conjugates, which were previously associated with restrictive urine (14). Together, these findings are consistent with catechol sulfates as biomarkers of urinary SCN ligand content.

**TABLE 1:** **Characteristics and parameters of pooled restrictive and permissive urines**

Parameter	Restrictive (mean ± S.D.)	Permissive (mean ± S.D.)	*p* value
Age	36 ± 9	34 ± 7	0.7402
pH	6.87 ± 0.24	5.74 ± 0.61	0.0136
Specific gravity	1.016 ± 0.014	1.008 ± 0.003	0.3033
SCN activity*^a^*	7.00 ± 1.79	−0.05 ± 0.13	0.0002

*^a^* SCN activity, as determined in Ref. 14, is equal to the log-scaled ratio of WT UTI89 growth to UTI89Δ*entB* growth in the presence of 1.5 μm SCN.

**TABLE 2:** **GC-MS metabolomics identifies candidate ligands enriched in DSF-positive fractions** R, restrictive; P, permissive.

Metabolite GC-MS identification	Retention time	Restrictive *vs*. permissive (approximate peak height ratio)	Standard obtained
	*min*		
Dihydroxy benzyl alcohol*^a^*	13.7	R only	
Caffeic acid*^a^*	18.9	R only	Yes
4-hydroxybenzaldehyde	9.92	R only	Yes
3-(3-Hydroxyphenyl)-3-hydroxypropanoic acid	15.5	33.1	
Indole-3-acetic acid	16.6	20.3	
Propyl gallate*^a^*	17.2	16.7	Yes
Pyrogallol*^a^*	11.9	16	Yes
3,4-Dihydroxyhydrocinnamic acid*^a^*	16.4	14.7	Yes
*trans*-Ferulic acid	17.7	11	Yes
3-Methoxy-4-hydroxybenzoic acid*^a^*	14.5	8.9	
Furoylglycine	13.4	7.7	Yes
4-Hydroxyhippuric acid*^a^*	19.1	2.4	
α-Hydroxyphenylacetic acid	11.1	2.3	
4-Hydroxyphenylacetic acid	13.1	1.5	Yes
3-Hydroxybenzoic acid	13	1.5	Yes
3-Hydroxyphenylacetic acid	12.7	1.3	
Methylcatechol*^a^*	10	1	Yes*^b^*
5-Hydroxyindole	13.3	P only	Yes

*^a^* Contains catechol moiety; may depend on isomer for some chemicals.

*^b^* Standards were obtained for both 3- and 4-methylcatechol isomers.

##### Restrictive Urines Possess Higher SCN Ligand Concentrations

**FIGURE 5.**
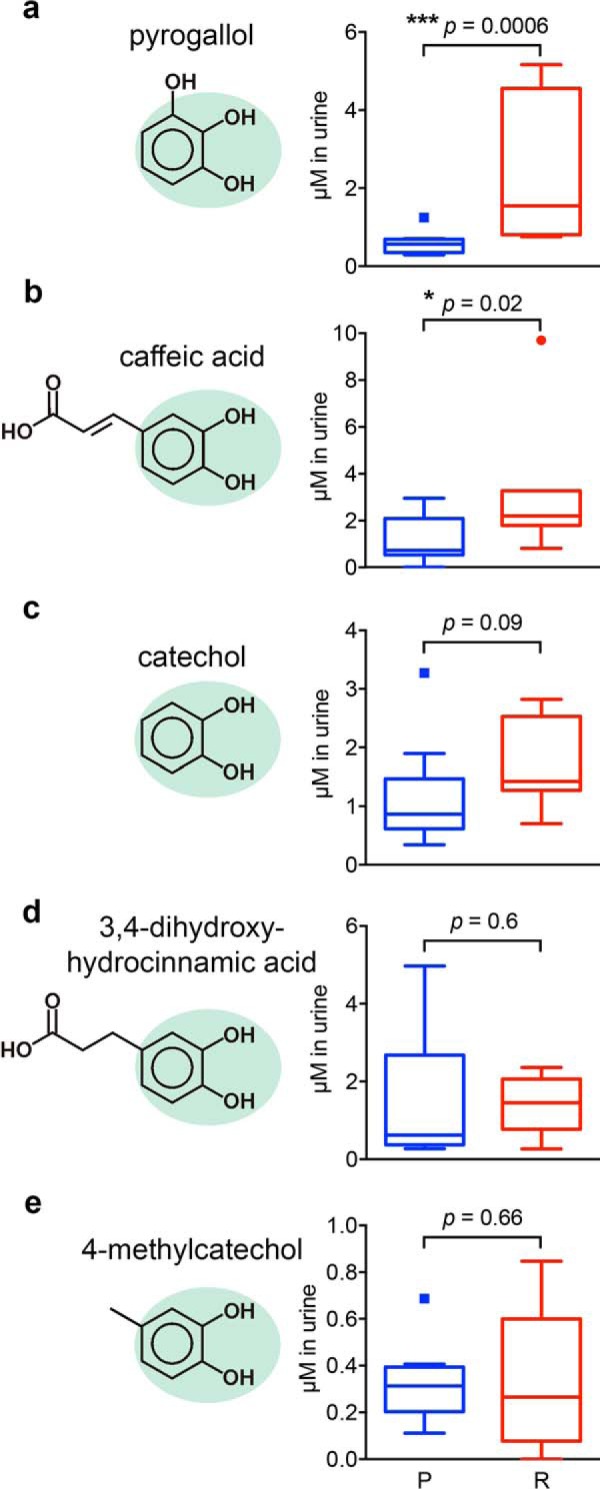
**Restrictive urines have improved correlation with urinary ligand concentrations.** We compared SCN ligand concentrations by GC-MS in restrictive (*R*; *red boxes*; *n* = 8) *versus* permissive urines (*P*; *blue boxes*; *n* = 9). Here, molar concentrations were measured in whole urine following urease treatment. Urines were spiked with the exometabolite 4FSA, and peak area ratios were compared with standard curves prepared with pure standard of each metabolite and 4FSA. Data are shown as *Tukey box-and-whisker plots*. Pyrogallol and caffeic acid were found to be significantly (*p* = 0.0006 and *p* = 0.02, respectively) elevated in restrictive samples (*a* and *b*). Catechol trended toward significance (*p* = 0.09) (*c*), whereas 3,4-dihydroxyhydrocinnamic acid (*d*) and 4-methylcatechol (*e*) were not consistently associated with restrictive or permissive urines in these samples. *, *p* < 0.05; ***, *p* < 0.001; Mann-Whitney *U* test.

**FIGURE 6.**
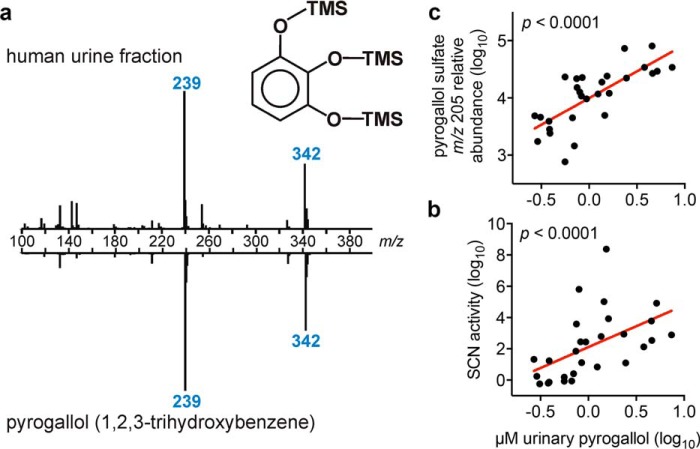
**Urinary pyrogallol is significantly correlated with SCN antimicrobial activity.** Urinary pyrogallol concentration, as confirmed by GC-MS (*a*), was found to increase significantly (*p* < 0.0001, Pearson correlation) with increasing SCN antimicrobial activity as defined in a previous study (14) (*b*). Furthermore, elevated molar concentrations of pyrogallol were also significantly correlated (*p* < 0.0001, Pearson correlation) with sulfated pyrogallol relative abundance as detected by LC-MS/MS (*c*). Each *point* represents data from a single donor urine specimen. Linear regression of log-transformed values (*red lines*) yielded *r* = 0.52 and slope = 2.68 ± 0.87 (*b*) and *r* = 0.74 and slope = 0.93 ± 0.17 (*c*).

To determine whether restrictive urine contains higher SCN ligand concentrations, we used GC-MS to quantify unconjugated pyrogallol, caffeic acid, 3,4-dihydroxyhydrocinnamic acid, 4-methylcatechol, and unsubstituted catechol (1,2-dihydroxybenzene). Propyl gallate and 3-methylcatechol were not reliably detected in these specimens. In a cohort containing eight highly restrictive urine specimens representing the top 20% of SCN activity and nine permissive specimens, restrictive urines possessed significantly higher pyrogallol (*p* = 0.0006) and caffeic acid (*p* = 0.02) concentrations than permissive urines (Fig. 5, *a* and *b*). Catechol, 3,4-dihydroxyhydrocinnamic acid, and 4-methylcatechol were detectable in all samples but not significantly different (*p* > 0.05) between restrictive and permissive groups (Fig. 5, *c–e*). Pyrogallol concentrations were positively associated (*p* < 0.0001) with SCN activity as defined previously (14) (Fig. 6, *a* and *b*); free urinary pyrogallol concentrations were also positively associated (*p* < 0.0001) with the relative abundance of urinary pyrogallol sulfate, an SCN activity correlate previously discovered through metabolomic profiling (Fig. 6*c*) (14). These findings suggest that, although SCN ligands may be ubiquitous in human urine, specific SCN ligands such as pyrogallol may be critical determinants of SCN activity in the complex urinary environment.

##### SCN Ligands Potentiate Antibacterial Activity in a Defined Medium

**FIGURE 7.**
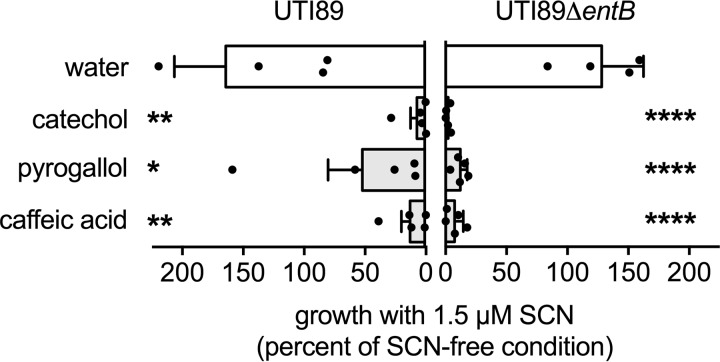
**SCN ligands potentiate the antimicrobial activity of SCN in a defined growth medium.** In M63 minimal medium, supplementation with SCN ligand catechol, pyrogallol, or caffeic acid (5 μm) limits *E. coli* UTI89 growth when combined with 1.5 μm SCN. Treatment with 1.5 μm SCN alone (SCN + water) does not limit bacterial growth in M63 minimal medium. Growth restriction is most dramatic in the enterobactin-deficient mutant, UTI89Δ*entB*, which is also not inhibited by SCN alone (water condition). Cultures were grown at 37 °C for 20 h and plated for cfu enumeration; *bars* indicate the mean of at least four independent cultures; *error bars* represent S.D. *, *p* < 0.05; **, *p* < 0.01; ****, *p* < 0.0001; one-way analysis of variance compared with water addition control.

To determine whether pyrogallol, caffeic acid, or catechol is sufficient to support SCN activity, we supplemented M63 minimal medium (23, 24) with concentrations of each compound that approximate levels found in human urine and measured *E. coli* growth. In medium only, SCN inhibited neither UTI89 nor its enterobactin-deficient mutant, UTI89Δ*entB* (Fig. 7). Addition of SCN ligands significantly inhibited growth of both strains with UTI89Δ*entB* inhibited to a greater degree (Fig. 7). A non-binding metabolite control, *trans*-ferulic acid (3-*O*-methylated caffeic acid), did not potentiate SCN activity nor did catechols when added to a nutritionally rich medium (Luria-Bertani (LB) broth) where iron availability does not limit growth (data not shown). These results support a direct role for urinary SCN ligand interactions in urinary SCN antibacterial activity.

##### Iron-Ligand-SCN Complex Detection with Native Mass Spectrometry

**FIGURE 8.**
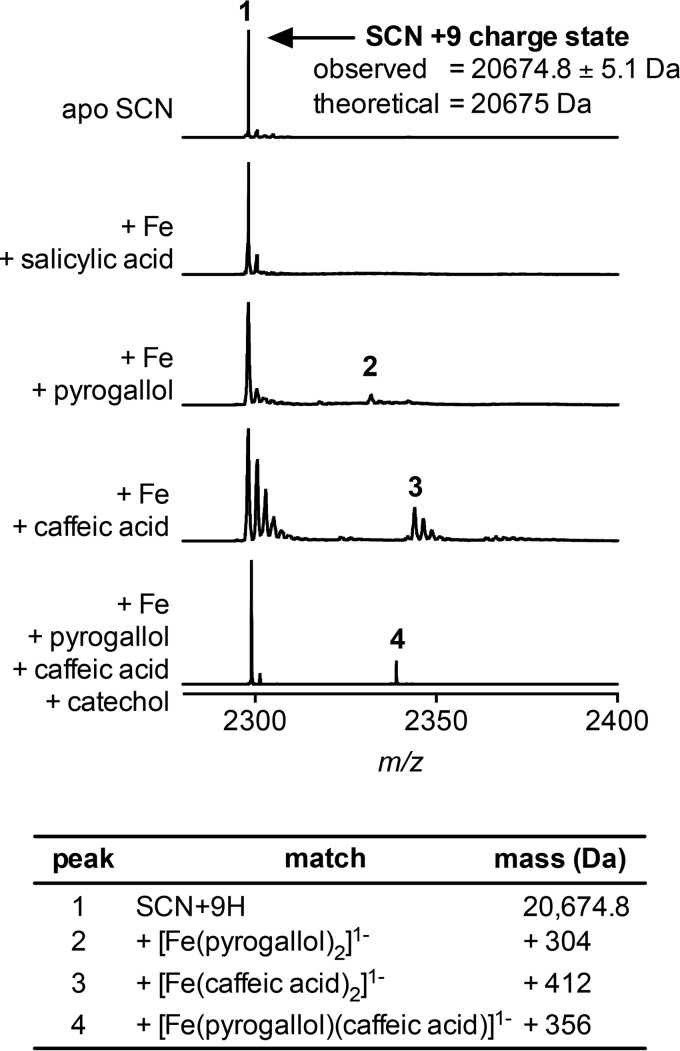
**SCN binds heterogeneous ligand complexes.** Native FTICR mass spectrometry detects SCN with an observed mass of 20675.0 ± 0.3 Da (20,675 Da calculated from sequence); data from the +9 charge state are shown here. Mixing apo-SCN (*peak 1*) with the urinary ligand pyrogallol or with caffeic acid yields new peaks that correspond to the mass of a 2:1 ligand-iron complex (*peaks 2* and *3*), whereas a non-binding control, salicylic acid, does not result in new peaks. Incubating SCN with an equimolar mixture of three urinary ligands (pyrogallol, caffeic acid, and catechol) produces a distinct peak at a mass corresponding to a [Fe(pyrogallol)(caffeic acid)]^1−^ complex (*peak 4*). Trailing peaks, most prominent in the caffeic acid sample, result from sodium (+22 Da) adducts common to native MS spectra. These results confirm that SCN does indeed bind heterogeneous complexes and suggest that these complexes may be preferentially bound over homogeneous complexes.

Because human urine contains multiple SCN ligands, we hypothesized that each SCN molecule may use a combination of different ligands in the three calyx pockets to chelate a ferric ion. To investigate this possibility, we used non-denaturing, native mass spectrometry to resolve calyx occupancy in the presence of multiple SCN ligands (25–28); a similar approach previously confirmed enterobactin binding to SCN (26). Using FTICR mass spectrometry, apo-SCN appeared as peaks at *m*/*z* 2585.4 and *m*/*z* 2298.2, matching the [SCN + 8H]^8+^ and [SCN + 9H]^9+^ charge state ions with an observed mass of 20,675.0 ± 0.3 Da (calculated mass from sequence, 20,675 Da; Fig. 8). Following equilibration with Fe(III) and ligands, new peaks corresponding to calyx occupancy by a 1:2 Fe(III)-pyrogallol or Fe(III)-caffeic acid complex were detected. No new peaks were observed with the non-binder salicylic acid (Fig. 8) (21). When SCN was exposed to a mixture of pyrogallol, caffeic acid, and catechol, a single new peak was evident (Fig. 8). This peak had a 356-Da mass shift, corresponding to a 1:1:1 Fe(III)-pyrogallol-caffeic acid complex. Although the SCN calyx possesses three binding pockets, the observed 2:1 complexes are consistent with the previously proposed stepwise ligand addition and high affinity of 2:1 ferric complexes (which still possess a delocalized negative charge) within the calyx (8, 29). To our knowledge, this is the first demonstration that SCN can use heterogeneous ligand combinations to chelate Fe(III). Together, these results further support a subgroup of urinary catechols as ferric ion-chelating SCN ligands and suggest that preferred ligand combinations may affect SCN antibacterial activity in urine.

## Discussion

Together, these results support a mechanistic connection between human metabolomic composition and the antibacterial activity of siderocalin. This connection emerges from combined biophysical and mass spectrometric analyses that resolved physiologically relevant SCN ligands from the highly complex and diverse human urinary metabolome. These ligands were sufficient to potentiate SCN activity in a defined medium, experimentally recapitulating their general relationship with SCN activity in urine. Together, these results suggest that the previously observed urinary antimicrobial metabolomic profiles (14) are associated with free catechols that act as iron-binding SCN cofactors. Among these catechols, pyrogallol is highly associated with urinary SCN activity and may combine with other catechols to sequester iron within the SCN calyx.

In this study, both enterobactin and SCN were observed to coexist during human UTIs (Fig. 1). Although SCN is able to form a tight complex with Fe(III)-enterobactin, enterobactin appears to paradoxically counteract the antibacterial activity of SCN in human urine where it can provide iron for bacterial use (14). This unexpected finding indicates that additional SCN interactions are relevant in the urinary context, including the formation of Fe(III)-catechol-SCN complexes in human urine as suggested by the present work. Specifically, urine can possess multiple SCN ligands, the relative amounts of these ligands are associated with SCN antibacterial activity, and SCN may favor specific ligand combinations when forming Fe(III) complexes. It is possible that there is a hierarchy of Fe(III)-catechol-SCN complexes with varying ability to withhold iron from microbes. Once enterobactin has competitively removed the iron from the Fe(III)-catechol-SCN complex, it is possible that residual occupancy of the calyx by catechols or other metabolites prevents SCN from sequestering ferric enterobactin, although this has not yet been demonstrated.

SCN ligands in human urine (pyrogallol, caffeic acid, catechol, and methylcatechol) have varied and complex origins. They are likely derived from plant-based polyphenolic precursors and may require metabolic processing by the intestinal microbiome prior to intestinal absorption (30–34). Following absorption, these compounds may be conjugated to sulfate or glucuronate in the liver, which would interfere with their ability to chelate ferric ions and serve as SCN ligands (8, 18, 35). Indeed, although sulfated catechols were initially associated with restrictive urine (14), this study identified only unconjugated catechols as urinary SCN ligands, consistent with previous reports on the binding capacities of SCN (8, 18). Although neurotransmitters such as dopamine and epinephrine are widely familiar human catechols, these molecules (and their related 3,4-dihydroxycatechol metabolites) do not readily bind SCN (8) and were not detected in the present study. Indeed, catechols with substituents that are *para* to one of the catechol alcohols appear to be poor SCN binders (18, 21, 36). Nevertheless, the native MS results raise the possibility that these poor binders might be effective SCN ligands in combination with other catechols. Mixed ligand complexes also may partially explain why some urine specimens support little SCN antimicrobial activity but contain some catechols; it may be certain combinations, in addition to favorable pH (14, 17), that enable SCN to restrict bacterial growth. A more detailed description of SCN binding will be necessary to resolve these possibilities.

Identifying new ligands and substrates for proteins and enzymes is a recurring challenge in biochemistry. Metabolomic associations motivated the approach described here, which sought new ligands in urine, a notoriously complex biological specimen, using a combination of classic chromatographic fractionation, biophysical screens, and mass spectrometry. In this study, GC-MS allowed us to accurately detect and quantify many small metabolites; however, it is possible there are additional ligands not readily detectible by this technique. Advances in native mass spectrometry allowed us to address binding degeneracy, the use of several distinct catechol substituents, between individual SCN complexes. Our chromatographic screen may therefore have missed SCN ligands that bind Fe(III) only in combination with other chemically distinct ligands. Native mass spectrometry may thus be an underused complement to crystallographic analyses in illuminating the complete physiologic binding behavior of a protein.

These results suggest new strategies for UTI prevention or treatment. Therapeutically manipulating urinary composition through diet and/or intestinal microbiome-based interventions may enhance SCN activity in the urinary tract (37). Elevating urinary pH would be expected to enhance the efficacy of these interventions as would administration of enterobactin biosynthesis inhibitors. Evaluating these interventions will require both more sophisticated experimental disease systems and non-toxic interventions amenable to direct clinical evaluation. The latter may be accomplished through standardized dietary intervention studies in conjunction with metabolomic profiling.

## Experimental Procedures

### 

#### 

##### Uncomplicated E. coli Cystitis Specimens

Urinary specimens were from a previously described study (14). The Institutional Review Board of the University of Washington approved all study protocols. All patients provided written informed consent for the sample collection and subsequent analyses. Clean catch midstream urine specimens were obtained from female patients with acute uncomplicated cystitis between 2008 and 2012 at the University of Washington Hall Health Primary Care Center. Women were eligible if they were aged 18–49 years, in good general health, and had fewer than 7 days of typical symptoms of acute cystitis defined using previously described criteria of dysuria, urinary frequency, and urinary urgency (38). SigmaFAST^TM^ protease inhibitor solution (Sigma) was added at 110 volume to freshly voided urines before clinical centrifugation, and the supernatant was frozen at −80 °C. Uropathogens in midstream urine were identified using standard methods. Urine specimens analyzed here were collected during subjects' initial visits with a diagnosis of *E. coli* acute uncomplicated cystitis with ≥10^5^ cfu/ml β-hemolytic isolate. Urinary SCN was quantified by ELISA as published previously (14).

##### Enterobactin Quantification

Urinary enterobactin was detected and identified using LC-MS/MS. Briefly, human *E. coli* cystitis urine samples were spiked 1:10 with ^13^C-labeled internal standard (23, 24), then filtered, and diluted 1:1 with water before LC-MS/MS analysis with a Shimadzu Prominence UFLC-coupled AB Sciex 4000 QTRAP mass spectrometer. Chromatography was conducted with a gradient of 0.1% formic acid (Fluka, Sigma) to 90% acetonitrile (EMD Millipore) + 0.1% formic acid using an Ascentis Express phenyl-hexyl column (100 × 2.1 mm, 2.7 μm; Supelco, Sigma). Negative electrospray ionization-derived [M − H]^−^ precursor-product ions were *m*/*z* 686 > 222 for linear enterobactin and *m*/*z* 716 > 232 for the corresponding ^13^C_30_ isotopologue internal standard. Peak area ratios were averaged for triplicate analyses.

##### Protein Purification

Human SCN protein (a kind gift from Roland Strong) was expressed on a pGEX4T vector in BL21 *E. coli* as described previously (5, 14). The human SCN calyx mutant K125A/K134A was expressed on a pET22b vector in BL21 *E. coli* and purified as described (8, 14). Protein purity was monitored by mass spectrometry and SDS-PAGE.

##### Medium Growth Assay

The defined medium used to test metabolite supplementation was M63 minimal salts medium supplemented with 0.2% glycerol and 10 μg/ml niacin. SCN activity assays were performed essentially as described previously (13, 14). Briefly, medium was inoculated with the uncomplicated cystitis isolate UTI89 or isogenic siderophore mutants (23) at 10^3^ cfu/ml from 4–6-h cultures in LB broth. Cultures were grown with 1.5 μm SCN or an equivalent volume of PBS. After 20 h, viable bacterial concentrations were determined by cfu enumeration. Where indicated, medium was supplemented with candidate metabolites at 5 μm.

##### Urine Fractionation

Healthy donor urines were collected as approved by the Washington University School of Medicine Institutional Review Board and described previously (14). To fractionate and concentrate urinary small molecules, we used a multistep chromatographic scheme. Four urines of either high or low SCN antimicrobial activity were pooled (2 ml each) and applied to Chrom P SPE cartridges (Supelco). The flow-through, water wash, and elutions with 50% and then 100% HPLC grade methanol (Sigma) were all collected and lyophilized to dryness. Each of the four preparations was then rehydrated in HPLC grade water (Sigma) and fractionated over a phenyl-hexyl Eternity column (250 × 4.6 mm, 5 μm; Supelco) using a water:methanol gradient on a BioLogic DuoFlow chromatography system (Bio-Rad). All fractions (∼290 total) were lyophilized to dryness and stored at −80 °C until analysis.

##### Differential Scanning Fluorimetry

To screen for SCN binding in urinary fractions, we measured thermal denaturation through DSF essentially as described (20, 39). Lyophilized urinary HPLC fractions were reconstituted in 100 μl of HPLC grade water, thus yielding a roughly 80-fold concentration from the original bulk urine. Fractions (10 μl) were added to a final concentration of 3 μm SCN in PBS with a 5× concentration of SYPRO Orange dye (Sigma) and either 3.2 μg/ml FeCl_3_ or an equal volume of water and allowed to equilibrate for 20 min. Using a CFX96 real time PCR machine (Bio-Rad), reactions were then heated in 96-well quantitative PCR plates (Bio-Rad) from 22 to 98 °C at 1 °C/min with plate reading at each increment. Melt transitions were monitored on the HEX channel and displayed as the derivative of relative fluorescence units with respect to temperature (d(RFU)/d*T*). *T_m_* values were calculated from the raw amplification curves using a Boltzmann regression in Prism version 7.0a (20). Fractions were also analyzed in the absence of protein as a negative control, SCN was incubated with ferric catechol (30 μm) as a positive control, and negative controls included protein plus iron and fractions without protein present. Interesting fractions were identified by a *T_m_* shift.

Preliminary compounds identified by GC-MS were also confirmed by DSF. These reactions were carried out as above but with a ferric complex (FeL_3_) concentration of 30 μm. This saturating ligand concentration is used to ensure all protein is in the bound form for the melt analysis as established (20). Chemicals were purchased from Sigma-Aldrich and prepared with FeCl_3_ or without (equal volume of PBS) at a 1:3 ratio of iron to ligand (FeL_3_). SCN melt curves were performed as above, and a positive DSF result was considered a *T_m_* shift or loss of the melt signal.

##### Fluorescence Quenching

We confirmed metabolite-SCN interactions by measuring binding of DSF-positive candidates by intrinsic tryptophan fluorescence quenching essentially as described (5, 8). SCN protein (500 nm) was mixed with a dilution series of ligand in the presence and absence of iron in PBS with 2% DMSO in black 96-well fluorescence plates (Corning). After 1-h equilibration, tryptophan fluorescence was monitored at excitation and emission wavelengths of 281 and 340 nm, respectively, on a Tecan Infinite 200 microplate reader at room temperature. Binding was monitored up to 48 h later with no loss of signal or notable change in fluorescence quenching patterns (data not shown).

##### Gas Chromatography-Mass Spectrometry

Fractions identified by DSF as containing SCN binding character were profiled by GC-MS. Briefly, *N*-methyl-*N*-trimethylsilyl trifluoroacetamide-derivatized samples were analyzed on an Agilent 7890A gas chromatograph interfaced to an Agilent 5975C mass spectrometer operated in the electron ionization mode; the source temperature, electron energy, and emission current were 230 °C, 70 eV, and 300 μA, respectively. GC was performed with an HP-5MS column (30 m, 0.25-mm inner diameter, 0.25-μm film coating; P. J. Cobert, St. Louis, MO) with a linear temperature gradient of 80–300 °C at 10 °C/min; the injector and transfer line temperatures were 250 °C. Fraction spectra were matched to the NIST11 Mass Spectral Library for chemical identification using automatic mass spectral deconvolution and identification system, and metabolites elevated in binding fractions compared with non-binding fractions were identified as potential ligand candidates and analyzed further by DSF and FQ.

To quantify SCN-binding metabolites by GC-MS, we produced calibration curves for each metabolite relative to the exometabolite 4-fluorosalicylic acid (4FSA) (14). Several metabolites could not be quantified because no standard was available, or the standard was not reliably detectable by GC-MS. Urine specimens (100 μl) were spiked with 200 pmol of 4FSA and treated with 2.4 units of urease (Sigma) for 40 min before quenching with 1 volume of methanol. The reaction was dried under nitrogen and derivatized as described above. GC-MS was performed in selected ion monitoring mode based on the two fragment ions for each candidate that were used for standard curves. Both fragment ions were monitored to assure correct identification. Molar concentrations were determined using the standard curve slope to correct the integrated peak area ratio of 4FSA and the analytes of interest.

##### Native Mass Spectrometry

SCN protein (30 μm) was prepared in 50 mm ammonium acetate (Sigma), pH 6.95. Where indicated, FeCl_3_ (50 μm) and SCN ligand compounds (60 μm for individual compounds; 100 μm total for mixed compounds) were added, equilibrated at room temperature for 2 h, and stored at 4 °C until analysis. For native electrospray ionization, a 10-μl sample was injected through a nanoES spray capillary (Thermo Scientific) interfaced with a Bruker Solarix 12T FTICR mass spectrometer (Bruker Daltonics, Bremen, Germany). The capillary voltage was 0.9–1.3 kV. The drying gas temperature was 30 °C; its flow was 1 liter/min. The in-source collision induced dissociation voltage was set to 70 V to help desolvation. The ion funnel rf amplitude was 300 V peak to peak, and the ion funnel voltages were 200 (funnel 1) and 18 V (funnel 2). rf values in all ion transmission regions were set to the lowest available value: multipole 1, 2 MHz; quadrupole, 1.4 MHz; and transfer line, 1 MHz. Ions accumulated for 500 ms in the rf hexapole ion trap before transmission to the infinity ICR trap. The time-of-flight was 1.3 ms for the protein-ligand ions. The source region pressure was 2.3 mbar; the quadrupole region pressure was 4.4 × 10^−6^ mbar, and the trap chamber pressure was 1.3 × 10^−9^ mbar. MS parameters were slightly modified for each individual sample to obtain an optimized signal. One to several hundred scans were averaged for each spectrum. External calibration was performed with electrospray ionization of cesium perfluoroheptanoic acetate to a maximum of *m*/*z* 8500.

##### Statistical Analyses

Data were analyzed in Prism version 6.0d (GraphPad). For parametric and non-parametric two-sample unpaired comparisons, we used *t* test and Mann-Whitney *U* test, respectively. To compare multiple groups *versus* control or each other, we used one-way analysis of variance. Where appropriate, post-tests were used to correct for multiple comparisons.

## Author Contributions

R. R. S.-C. and J. P. H. designed the studies and wrote the paper. J. R. C. and R. R. S.-C. performed and analyzed GC-MS experiments. C. D. M. and R. R. S.-C. performed and analyzed DSF and FQ on SCN. A. E. S. designed and conducted the study from which cystitis urines were acquired. W. C. performed native MS experiments. R. R. S.-C. performed all other experiments, analyzed data and results, and prepared the figures. All authors reviewed the results and approved the final version of the manuscript.

## Supplementary Material

Supplemental Data

## Footnotes

3 The abbreviations used are:

SCN: siderocalin
UTI: urinary tract infection
DSF: differential scanning fluorimetry
FQ: fluorescence quenching
FTICR: Fourier transform ion cyclotron resonance
2,3-DHBA: 2,3-dihydroxybenzoic acid
*T_m_*: melting temperature
SPE: solid phase extraction
4FSA: 4-fluorosalicylic acid
mbar: millibars.
