# Supplementary material for: Human Metabolome-derived Cofactors Are Required for the Antibacterial Activity of Siderocalin in Urine
Source: J Biol Chem. 2016 Oct 25;291(50):25901–10. doi: 10.1074/jbc.M116.759183 (PMC5207064; doi:10.1074/jbc.M116.759183)
Supplement: Supplemental Data [file supp_291_50_25901__index.html]

Human Metabolome-Derived Cofactors are Required for the Antibacterial Activity of Siderocalin in Urine — Human Metabolome-derived Cofactors Are Required for the Antibacterial Activity of Siderocalin in Urine — Human Metabolites Are Antibacterial Siderocalin Cofactors — Supplemental Data 

# Human Metabolome-derived Cofactors Are Required for the Antibacterial Activity of Siderocalin in Urine

## Supplemental Data

- Supplemental Data (.pdf, 1.8 MB) - Figures S1 and S2, containing extended differential scanning fluorimetry data.
