# Supplementary material for: Human Metabolome-derived Cofactors Are Required for the Antibacterial Activity of Siderocalin in Urine
Source: J Biol Chem. 2016 Oct 25;291(50):25901–10. doi: 10.1074/jbc.M116.759183 (PMC5207064; doi:10.1074/jbc.M116.759183)
Supplement: Supplemental Data [file 10.1074_M116.759183_jbc.M116.759183-1.pdf]

SUPPLEMENTAL DATA

Human Metabolome-Derived Cofactors are Required for the Antibacterial Activity of Siderocalin in Urine

Robin R. Shields-Cutler[†‡], Jan R. Crowley[§], Connelly D. Miller[†‡], Ann E. Stapleton[¥], Weidong Cui[£], and Jeffrey P. Henderson[†‡*]

[†]Division of Infectious Diseases, Department of Medicine, Washington University School of Medicine, St. Louis, MO.
[‡]Center for Women's Infectious Diseases Research, Washington University School of Medicine, St. Louis, MO.
[§]Department of Internal Medicine, Washington University School of Medicine, St. Louis, MO.
[¥]Department of Medicine, Division of Allergy and Infectious Diseases, University of Washington, Seattle, Washington.
[£]Department of Chemistry, Washington University in St. Louis, St. Louis, MO.

TABLE OF CONTENTS

Figure S1	pg. 2-3
Figure S2	pg. 4

*To whom correspondence should be addressed: Jeffrey P. Henderson, M.D., Ph.D., Division of Infectious Diseases, Washington University School of Medicine, 660 S. Euclid, St. Louis, MO 63110, USA, Tel: (314) 747-0240; Fax: (314) 454-8294; E-mail: jhenderson@DOM.wustl.edu

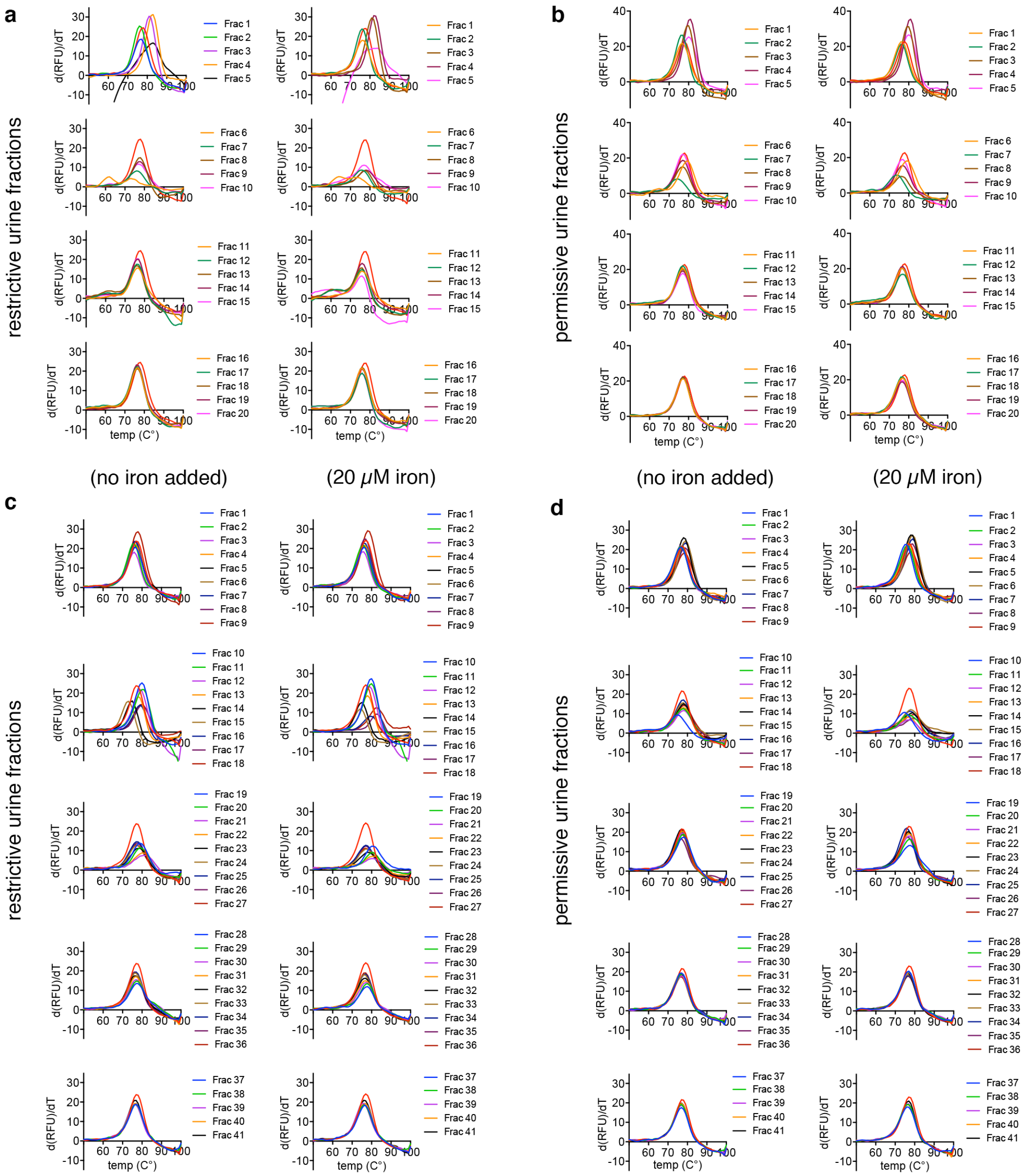
a
b
c
d
restrictive urine fractions
permissive urine fractions
d(RFU)/dT
temp (C°)
(no iron added)
(20 μM iron)

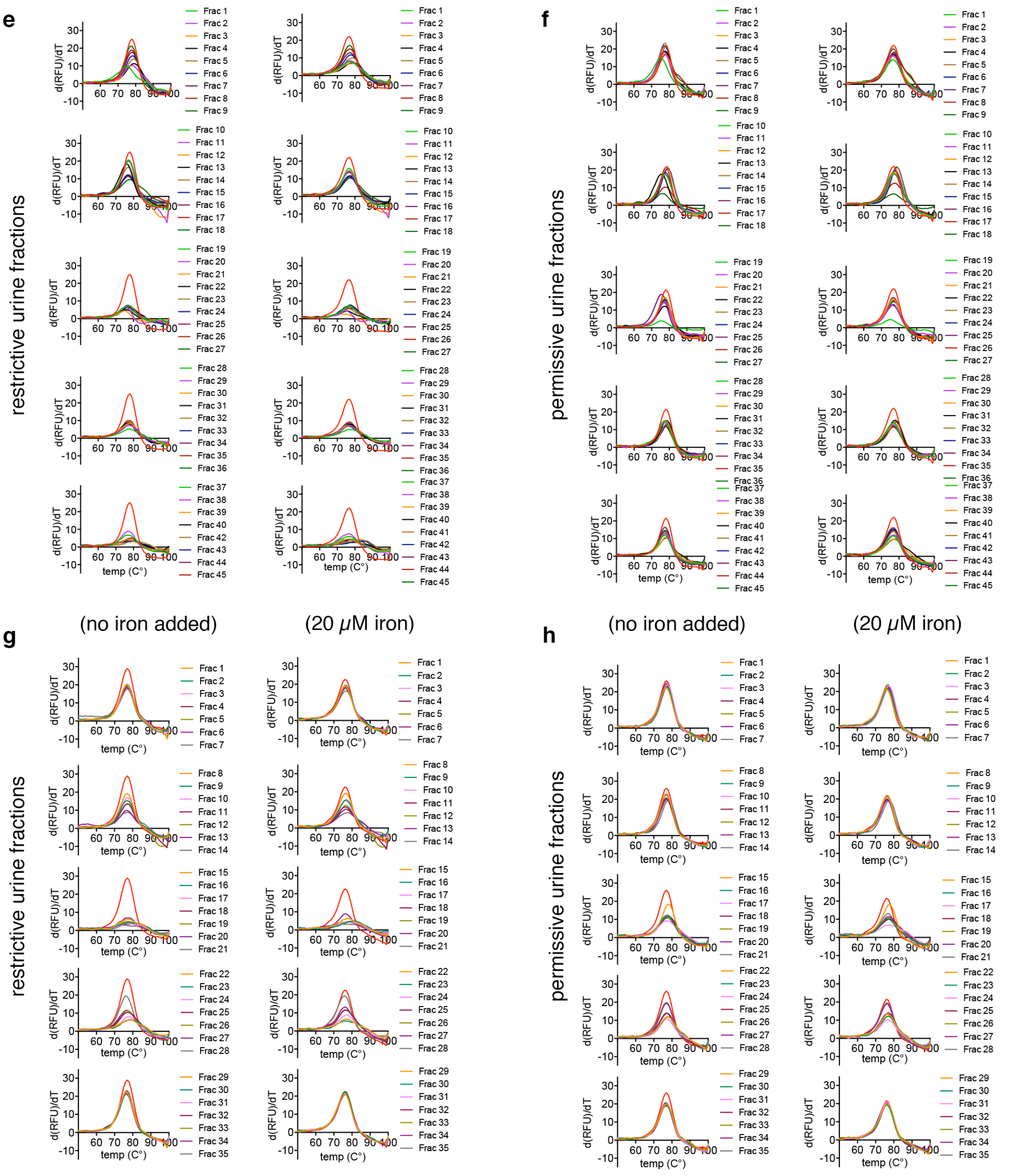

Figure S1. DSF screen identifies fractions with SCN-binding character. HPLC fractions from four solid-phase elutions were individually tested for SCN binding by DSF. Fractions were reconstituted in water and mixed with 3 μM SCN in PBS with or without an additional 20 μM iron as indicated. Plots are divided into subgroups of sequential fractions for visibility. Pooled restrictive (left column of panel) or permissive (right column of panel) urines (equal volumes of urine from four donors for each condition) were fractionated over Chrom P SPE and collected as the flow-through (**a**,**b**), water wash (**c**,**d**), 50% methanol elution (**e**,**f**), and 100% methanol elution (**g**,**h**). DSF was performed as described in the methods; plots are shown as the first derivative of the fluorescence signal. In each plot, SCN protein alone is shown as a red line for comparison.

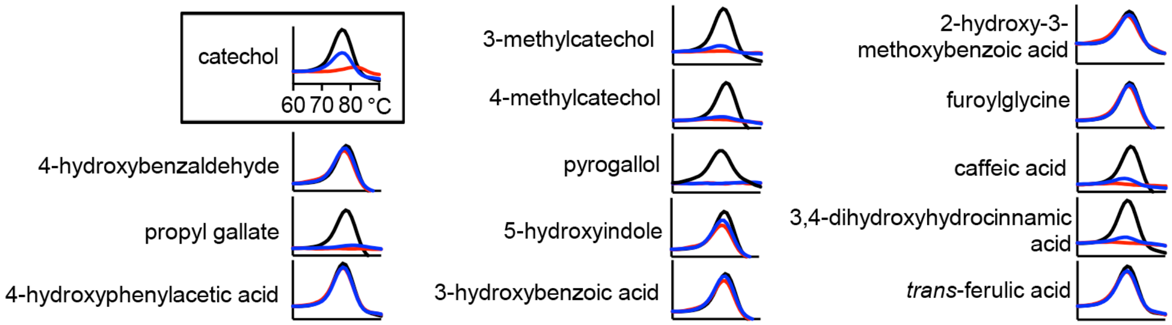

Figure S2. Urinary aryl alcohols perturb SCN thermal stability by DSF. Candidate metabolites and known SCN ligands were obtained commercially and tested for SCN binding in the presence and absence of iron. Compounds were mixed in a 3:1 molar ratio with $FeCl_3$ (in red) or an equivalent volume of PBS (in blue) and added to SCN (3 μM) before measuring thermal stability by DSF. Plots are shown as the first derivative of the fluorescence signal (arbitrary units) with respect to temperature; black lines show SCN with buffer alone. Based on the behavior of a known ligand (catechol), T_M shifts or signal ablation were considered positive results to be analyzed further. Axes for all plots are as shown in the catechol plot.
